# Response to immunotherapy in KRAS G12C mutated NSCLC: a single-centre retrospective observational study

**DOI:** 10.18632/oncotarget.28230

**Published:** 2022-05-11

**Authors:** Carolina Sciortino, Valentina Viglialoro, Massimo Nucci, Mariam Grazia Polito, Enrico Cortesi, Alain Gelibter, Paola Gazzaniga, Chiara Nicolazzo, Marco Siringo, Salvatore Caponnetto

**Affiliations:** ^1^Department of Radiology, Oncology and Pathology, Policlinico Umberto I, Sapienza University of Rome, Rome, Italy; ^2^Medical Oncology Unit B, Department of Radiological Oncological and Pathological Sciences, University La Sapienza, Rome, Italy; ^3^Medical Oncology Unit B, Department of Radiology, Oncology and Pathology, Policlinico Umberto I, Sapienza University of Rome, Rome, Italy; ^4^Medical Oncology Unit, Department of Clinical and Molecular Medicine, Sapienza Università di Roma, Rome, Italy

**Keywords:** NSCLC, immunotherapy, KRAS G12C mutation, liquid biopsy

## Abstract

Background: Non-small cell lung cancer is the leading cause of cancer death worldwide. New strategies in molecular therapies are being explored to detect and target genetic mutations in NSCLC. Therefore, it is also important to understand the interaction between these mutations and other therapies. This study focuses on possible correlations between the KRAS-G12C mutation and response of patients treated with immunotherapy.

Methods: Twenty-two patients with stage IV NSCLC undergoing immunotherapy were divided into two groups treated with first- and second-line therapy, respectively. KRAS-G12C mutation was detected by liquid biopsy Idylla KRAS assay.

Results: In first-line treated patients, there was no significant increase in PFS in patients with the KRAS mutation (20 months versus 14.5 months, HR = 1.31; CI 95% = 0.25–6.71; *p* value = 0.76) and no difference in OS (OS = 21 months, HR = 1; CI 95% = 0.17–6.2; *p* value > 0.99). In the second group, KRAS G12C mutated patients had a median PFS of 23 months compared with a median PFS of only 5 months among nonmutated patients (HR = 3.28; CI 95% = 0.86–12.5; *p* value = 0.03).

Conclusion: The results of this study do not reveal a clear correlation between mutation and response to immunotherapy. The mechanism regulating immune system activity in the tumor microenvironment remains unclear.

## INTRODUCTION

NSCLC (non-small cell lung cancer) accounts for nearly 70% of all lung cancers. The asymptomatic progression of the disease has made NSCLC the leading cause of cancer death worldwide. Most patients are diagnosed at advanced stages, so the development of a new line of therapy is essential to have a better life expectancy outcome in NSCLC. The median overall survival for patients with pathologic stage IV is 17 months. With current therapies, 9–18% of patients have a response to even third-line therapies with a median progression-free survival of 2.5–4.0 months [1, 2]. These responses are due to the chemotherapeutic and immunotherapeutic options we have in the treatment of NSCLC. The transition of therapies in metastatic NSCLC is progressing toward personalized treatments that provide more precise targets and allow for appropriate patient selection.

Thus, it is necessary to consider the heterogeneous biology of this cancer, and how different settings of driver mutations have different prognostic implications. KRAS mutations are linked to poor survival benefit in NSCLC, confirmed by a meta-analysis by Min Ying and Xiao-Xia Zhu [3]. In KRAS mutant NSCLC, a possible superior efficacy of ICIs has been observed, especially with anti-PD-1/PD-L1, but the relationship remains unclear [4, 5]. These uncertainties may be due to the different amino acid exchange we have in KRAS as driver mutations.

### Structure and function of KRAS

The Kristen rat sarcoma viral oncogene homolog (KRAS) is mutated in approximately 20% of lung adenocarcinomas and even more in squamous cell carcinoma [6, 7]. KRAS-mutant tumors represent the most frequently targeted molecular subtypes. But KRAS mutant tumors themselves might be composed of a heterogeneous set of diseases. Among the different KRAS mutations G12C occurs in 13% of NCLCs [8, 9]. This amino acid exchange mutation from glycine to cysteine at position 12 (G12C) leads to the active form of the protein, which is predominantly GTP-bound. This conformation results in enhanced survival and proliferation in cancer cells [9, 10]. This is likely due to several downstream pathways of RAS. Raf is the first protein in the MAPK (mitogen-activated protein kinase pathway), which activates MEK that promotes the activation of ERK (extracellular signal-regulated kinase). ERK translocates to the nucleus, stimulates proliferation and survival, playing an essential role in tumorigenesis [11].

PI3K also plays a key role with the activation of AKT, leading to the phosphorylation of several substrates such as mTOR, FOXO and NF-κB.

All these factors, in addition to stimulating cell cycle progression and survival, promote cellular metabolic switch, cell migration, and resistance to apoptosis [12, 13]. The active form of KRAS activates transduction of all these pathways. Cancer cells are entirely dependent on KRAS mutation which makes KRAS an oncogene.

### KRAS and PD-1/PD-L1 in NSCLC

KRAS-mutant NSCLC is the typical smoking-associated lung cancer and remarkably a tumor with a high mutational burden [14, 15].

Some studies report elevated PD-L1 expression through activation of downstream pathways of KRAS, including AKT-mTOR and MAPK signaling, and also increased expression induced by tobacco smoke. These factors might be responsible for increased T-cell infiltration in this type of tumor. We might speculate that KRAS is vulnerable to anti-PD-1/PD-L1 immunotherapy, but this possibility is not yet clearly demonstrated [4, 5].

## RESULTS

Retrospective analysis was started by dividing the two groups of patients into first- and second-line, then molecular biology was assessed and we identified KRAS G12C patients in both groups and progression-free survival and overall survival of each patient.

We drew up our data more within the KRAS G12C mutation, also considering the variability of different mutations within the KRAS family cause of the heterogeneity of some outcomes based on specific mutations.

**Figure 1:**
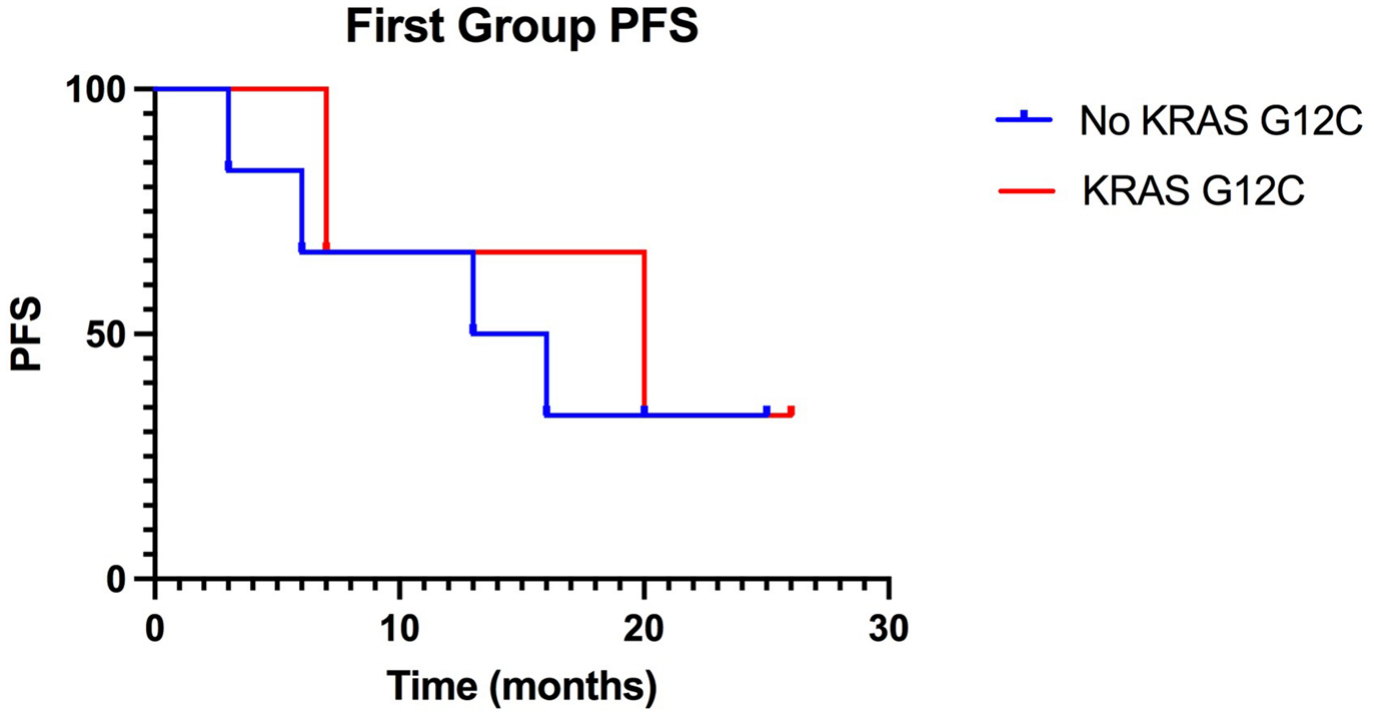
Kaplan-Meier of PFS in the first group, treated with ICIs in first-line: KRAS-G12C mutated patients have a median PFS of 20 months compared to 14,5 months PFS of non-KRAS-G12C mutated patients. (*p* value: 0.76).

In our study, we focused on the same type of correlation between specific mutation and treatment with ICIs targeting the PD-1/PD-L1 pathway. Within the first group (first-line treatment with ICIs), there was no significant increase in PFS in patients with the KRAS mutation. The median PFS obtained for KRAS mutant patients was 20 months compared with the median PFS in NO KRAS G12C of 14.5 months, with low statistical significance (Figure 1, Table 1). OS was not found to be different, finding in both KRAS mutant and NO KRAS G12C patients an OS of 21 months with thus an HR equal to 1.

**Table 1:** First group results

	PFS median	HR	IC 95%	*P* value
**NO KRAS G12C**	14.5	1.31	0.25–6.71	0.76
**KRAS G12C**	20	0.77	0.15–3.94	
	**OS median**			
**NO KRAS G12C**	21	1	0.09–11.3	>0.99
**KRAS G12C**	21	1	0.17–6.2	

**Figure 2:**
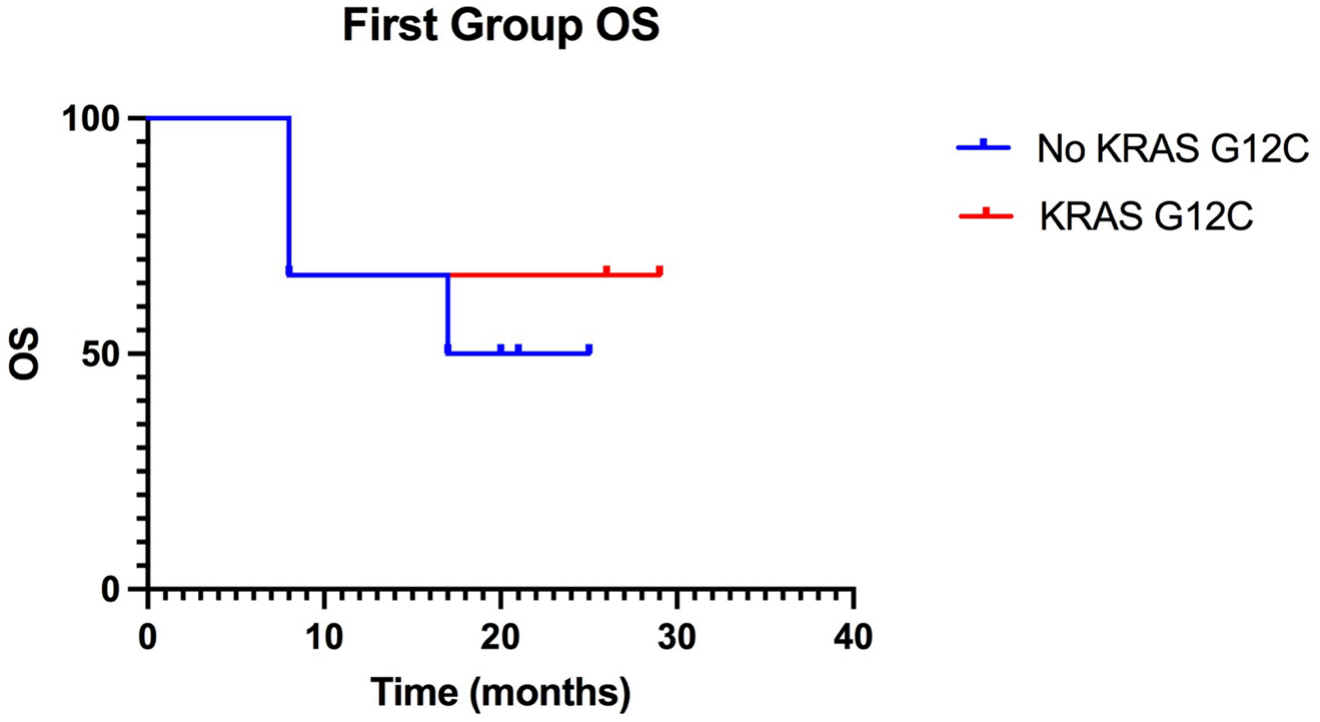
Kaplan-Meier of OS in the first group, treated with ICIs in first-line, the curves, of KRAS-G12C mutated patients and of non-KRAS-G12C mutated, overlap. (*p* value: >0.99).

As seen in the graph in Figure 2, the Kaplan-Meier survival curves tended to overlap between mutants and NO KRAS G12C, indicating the lack of differences between KRAS G12C and NO KRAS G12C patients.

In the second group, by analyzing the data shown in Table 2, it was possible to show that there was a greater response in patients with KRAS G12C mutation compared to NO KRAS G12C.

**Table 2:** Second group results

	PFS median	HR	IC95%	*P* value
**NO KRAS G12C**	5	3.28	0.86–12.5	0.03
**KRAS G12C**	23	0.30	0.08–1.16	
	**OS median**			
**NO KRAS G12C**	6.5	2.17	0.56–8.44	0.22
**KRAS G12C**	23	0.45	0.19–1.79	

**Figure 3:**
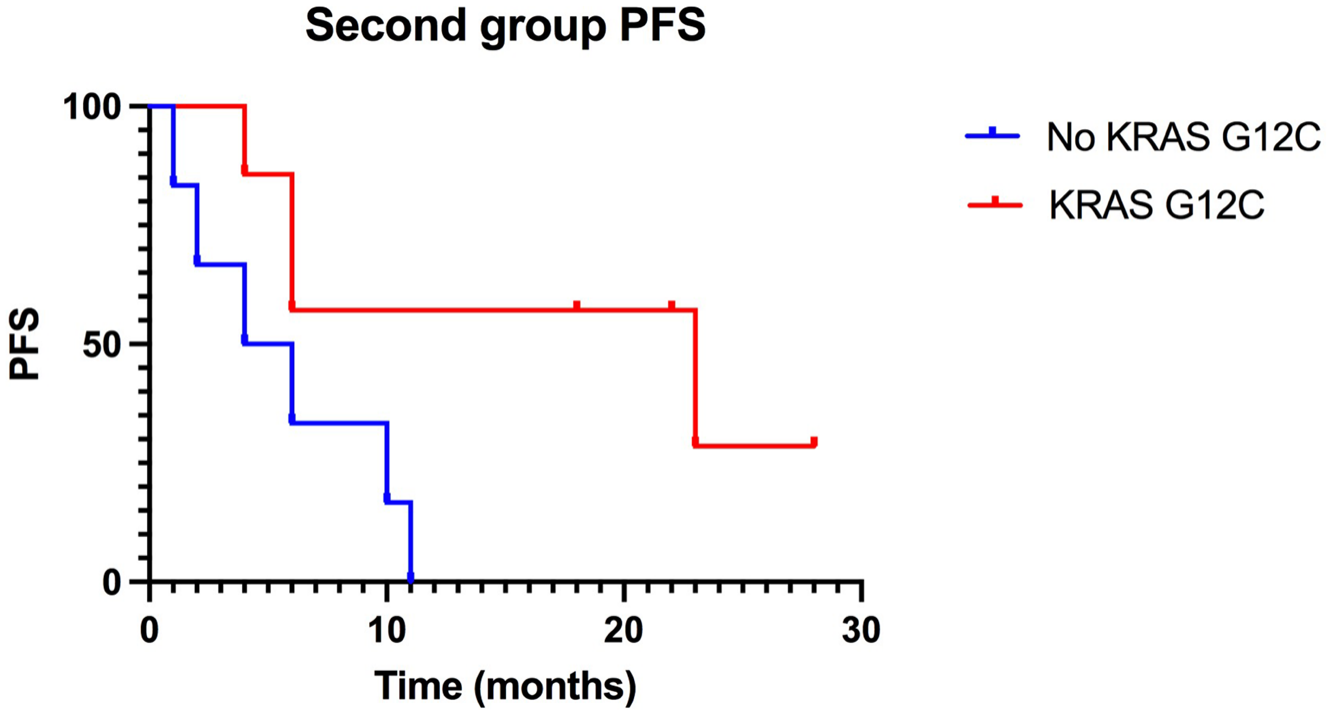
Kaplan-Meier of PFS in the second group, treated with ICIs in second-line: KRAS-G12C mutated patients have a median PFS of 23 months compared to 5 months PFS of non-KRAS-G12C mutated patients. (*p*-value: 0.03)

**Figure 4:**
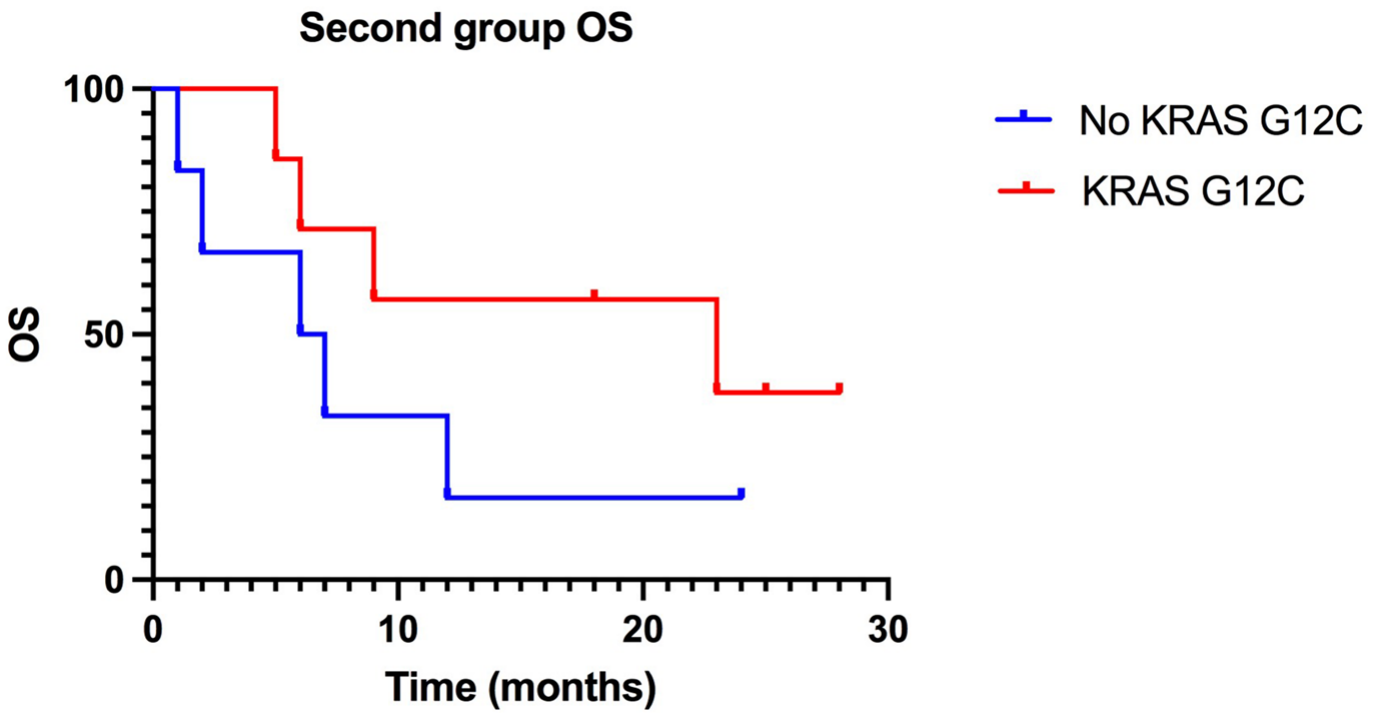
Kaplan-Meier of OS in the second group, treated with ICIs in second-line: difference between KRAS-G12C mutated patients 2019; OS (23 months) and NO KRAS G12C mutated patients 2019; OS (6, 5 months) is statistically significant. (*P* value: 0, 22)

Within the patients with KRAS G12C mutation there was a median PFS of 23 months compared to a median PFS of only 5 months in the NO KRAS G12C group. This difference in trend was statistically significant with a *P* value = 0.03 (Figure 3, Table 2). Our results reflect the literature in terms of PFS, identifying an improvement in patients with the mutation. The same disparity was found in terms of OS (Figure 4).

Also reported in Table 2, we have a median OS in KRAS G12C of 23 months versus one of 6.5 for NO KRAS G12C patients, suggesting an improvement not only in response but also in survival for these patients, but without the statistical significance previously found for PFS (*P* value = 0.22).

## DISCUSSION

The retrospective study was conducted in 22 patients diagnosed with stage IV NSCLC, selected from those who were treated with ICIs specifically directed against PD-1/PD-L1 and in whom the presence of the KRAS G12C mutation was sought.

The aim was to identify a possible relationship between this mutation and a greater benefit to treatment with anti PD-1/PD-L1, also showing two different trends between first and second line treatment with ICIs in NSCLC.

In literature, we found that KRAS-dependent tumors have a worse prognosis than wild-type patients, with a reduction in both disease-free interval (HR: 1.33; 95% CI 1.17–1.51) and overall survival (HR: 1.39; 95% CI 1.23–1.56).

At the same time, however, immunotherapy treatment has been found in several studies to induce benefit within the same KRAS mutated patients [5, 16, 17].

Among these, a 2019 study reports the results of nine other clinical trials, involving a total of 1716 patients with NSCLC, regarding the response to anti PD-1/PD-L1 treatment.

The Objective Response Rate (ORR) in patients with KRAS mutations was significantly higher than in patients with wild-type genotype, with good statistical significance, confirming the possible improved response (OR = 1.51; 95% CI: 1.17–1.96; *P* value = 0.002).

The same study also showed that the 6-month survival rate was higher in the KRAS-positive group than in the KRAS-negative group, although of uncertain statistical significance (OR = 1.34; 95% CI: 0.84–2.12; *P* = 0.22) demonstrating greater sensitivity to treatment and possible survival gain (Liu C, 2020). In a further study, it was reported that in a total of 705 patients receiving ICI monotherapy, the median overall survival for mutated KRAS (363 patients) was 21.1 months compared with 13.6 in 342 patients with wild-type KRAS genotype (HR = 0.77, CI95% = 0.61–0.98 *P* value = 0.03), supporting the hypothesis of a mechanism underlying an improved immunotherapeutic response of KRAS [16].

In our study, analyzing the first line, a difference in PFS was found although not statistically significant, while there is a substantial overlap in OS six patients carrying the KRAS G12C mutation compared to those who did not have the same mutation. This result is not statistically significant given the small number of patients, which limits the study itself.

The discourse is different for the second-line treatment group in which an improvement in survival of patients with KRAS G12C was identified, even more pronounced with regard to PFS.

This result, also limited by the small sample size, coincides with the literature regarding KRAS mutations and their response to immunotherapy. This suggests an increased susceptibility to treatment with ICIs induced by the presence of the KRAS mutation.

The detection of increased susceptibility for specific mutations could be useful for comparison in terms of efficacy of future target therapies also for KRAS G12C, already in development, such as MRTX849 (Adagrisib) or AMG510 (Sotorasib) [18].

Regarding the use of this target therapy, its relationship with current ICIs-based therapies should be studied, for possible different lines of treatment. In particular, in the study by D. S. Hong et al. the results concern 59 patients with NSCLC documenting that 89.8% had been previously treated with ICIs before undergoing the new treatment [19].

Similarly, in another study, therapeutic strategies for NSCLC are analyzed in detail, including specific treatment with antiPD-1/PD-L1 and KRAS G12C ICIs, describing initial treatment with ICIs [12]. From the results of the literature, we envision a possible therapeutic sequence in the setting of KRAS G12C mutated NSCLC that will include a first line based on immunotherapy and a subsequent line of treatment with Sotorasib.

Unfortunately, our study has several limitations within which our results need to be interpreted. The single-center retrospective design and the low number of patients may have prevented us from finding a significant correlation between response to immunotherapy and KRAS G12C mutation in NSCLC. Considering the potential clinical impact of finding a clear correlation, conclusions drawn from the study should be evaluated in the light of further research.

## MATERIALS AND METHODS

Response was assessed according to national guidelines: PFS was defined as the time in months from the date of the first ICI dose to the first documented progression, OS as the time in months from the first ICI dose to death from any cause.

In this retrospective study, clinical data were extracted from the medical records of 22 elderly patients with stage IV NSCLC (median age 70.6), from October 2018 to April 2021, treated with ICI and with available molecular analysis.

They were divided into two groups: Group A, who received first-line chemotherapy and then immunotherapy; Group B, who received immunotherapy directly. Group A consisted of four female and nine male patients. In the first line we record several chemotherapeutic options mainly based on Carboplatin. Developing disease progression (PD), they are undergoing ICI (immune checkpoint inhibitors). They underwent Atezolizumab (9 patients) and Nivolumab (3 patients), monoclonal antibodies against PD-L1 and PD-1, respectively. Group B consisted of three female and six male patients. They underwent immunotherapy based on another ICI, Pembrolizumab, a monoclonal antibody that has the same target as Nivolumab: PD-1.

### Detection of mutations

Patients in both groups underwent liquid biopsy for detection of the KRAS G12C mutation (Table 3, Table 4). Baseline mutation panels were compared with conventional biopsy tissue (4 missing biopsy reports because of clinical conditions that did not allow invasive analysis as traditional biopsy in all patients).

**Table 3:** First group molecular biology

Patient	Tissue panel	Plasma KRAS (Idylla)
1	KRAS G12C (22%)	KRAS G12C
2	Missing data	wt
3	KRAS G12D (11.5%) p53 (5.4%)	KRAS G12D
4	KRAS G12C (15%)	KRAS G12C
5	SMAD4 (18%) STK11, p53R273c	wt
6	KRAS G12D (12%)	KRAS G12D
7	STK11(27,7%)	wt
8	KRAS G12D (25%)	KRAS G12D
9	Missing data	KRAS G12C

**Table 4:** Second group molecular biology

Patient	Tissue panel	Plasma KRAS (Idylla)
1	ERBB4 (45,4%) KRAS G12C(0,23%)	KRAS G12C
2	Missing data	KRAS G12C
3	TP53 (9%)	wt
4	KRAS G13D (22%)	KRAS G12D
5	P53 p.R248Q (6%)	wt
6	TP53 (8%); KRAS G12C (11%)	KRAS G12C
7	TP53	wt
8	Missing data	KRAS G12C
9	BRAF p.V600E (10%) KRAS G12D (22%)	KRAS G12D
10	Missing data	KRASG12C
11	KRAS G12C	KRAS G12C
12	STK11 (95%) CTNNB1; (33,7%) KRAS G12C (0.82%)	KRAS G12C
13	KRAS Q61K (25,2%)	KRAS Q61K

The patient’s plasma was analyzed with the IdyllaTM ctKRAS mutation assay.

This is an automated system that can identify 21 different mutations in exons 2, 3, and 4 of KRAS with high sensitivity and specificity (>95% compared with traditional systems) [20].

Based on the increased efficacy and recent discovery of MRTX849, a selective and covalent inhibitor of KRAS G12C, we focused on this specific mutation. MRTX849, called Adagrasib, selectively modifies the mutant cysteine residue in GDP-bound KRAS G12C and inhibits GTP binding and downstream KRAS-dependent signaling. The drug inhibits the *in vivo* growth of multiple KRAS G12Cs [18].

In the first group, the presence of the mutation was detected in three of the eight patients. In the second group, positivity for KRAS G12C was found in seven patients. Starting from the detection within the same groups, the efficacy of treatment with ICI was compared in terms of PFS and OS between patients positive for the specific mutation and those who did not harbor it (Tables 5 and 6).

**Table 5:** First group PFS and OS

Patient	KRAS G12C	PFS (months)	OS (months)
1	+	26+	26+
2	−	20+	20+
3	−	16	17
4	+	20	29+
5	−	25+	25+
6	−	13	21
7	−	3	8
8	−	6	6
9	+	7	8

**Table 6:** Second group PFS and OS

Patient	KRAS G12C	PFS (months)	OS (months)
1	+	6	6
2	+	6	9
3	−	4	6
4	−	10	12
5	−	11	24+
6	+	18+	18+
7	−	6	7
8	+	23	23
9	−	2	2
10	+	28+	28+
11	+	22+	22+
12	+	4	5
13	−	1	1

### Statistical analysis

All data were analyzed using Graphpad Prism 9.0.0 software.

We developed the Kaplan-Meier survival curves and also compared these results according to the Log Rank test.

The statistical significance sets at a *P* < 0.05. Taking in consideration patient’s death or loss at follow-up, the OS was calculated. PFS is assessed from the start of treatment with ICI until a disease progression is documented.

## CONCLUSIONS

We propose this study as a future starting point for a more substantial analysis of the correlation between response to immunotherapy and specific KRAS mutations, also exploiting liquid biopsy as a method to monitor the progress of these mutations during the treatment itself and the changes that may be evidenced at the time of disease progression. The mechanism of the tumor microenvironment increasing the expression of tumor antigens in the presence of KRAS mutations remains unclear, although there is much evidence of increased leukocyte infiltrate in these tumors with an increased lymphocyte/cell, neutrophil ratio, indicating a better immunotherapeutic response [4]. Indeed, the trend of gene expression of tumor cells during treatments should be studied in order to also recognize how the activity of the immune system influences the different pools of mutations present in the tumor, with and without the help of treatment based on antiPD-1/PD-L1 ICIs, providing us with information about the selective pressure our body has on tumor cells and the different mutations, including KRAS G12C.

## Abbreviations

NSCLC: non-small lung cancer
KRAS: Kristen rat sarcoma oncogene
ICI: immune checkpoint inhibitor
PD-1: programmed death 1
PD-L1: programmed death ligand 1
MAPK: mitogen-activated protein kinase
ERK: extracellular signal-regulated kinase
mTOR: mechanistic target of rapamycin
FOXO: forkhead box O
NF-κB: nuclear factor kappa-light-chain-enhancer of activated B cells
PFS: progression-free survival
OS: overall survival
